# The Prevalence and Associated Death of Ventricular Arrhythmia and Sudden Cardiac Death in Hospitalized Patients With COVID-19: A Systematic Review and Meta-Analysis

**DOI:** 10.3389/fcvm.2021.795750

**Published:** 2022-01-21

**Authors:** Ziqi Tan, Shan Huang, Kaibo Mei, Menglu Liu, Jianyong Ma, Yuan Jiang, Wengen Zhu, Peng Yu, Xiao Liu

**Affiliations:** ^1^Department of Endocrine, The Second Affiliated Hospital of Nanchang University, Nanchang, China; ^2^Department of Psychiatry, The Third People's Hospital of Ganzhou, Ganzhou, China; ^3^Department of Anesthesiology, The People's Hospital of Shangrao, Shangrao, China; ^4^Department of Cardiology, The Seventh People's Hospital of Zhengzhou, Zhengzhou, China; ^5^Department of Pharmacology and Systems Physiology, University of Cincinnati College of Medicine, Cincinnati, OH, United States; ^6^Department of Pharmacy, Harbin Medical University, Harbin, China; ^7^Department of Cardiology, The First Affiliated Hospital of Sun Yat-sen University, Guangzhou, China; ^8^Department of Cardiology, The Second Affiliated Hospital of Nanchang University, Nanchang, China

**Keywords:** arrhythmia, ventricular arrhythmia, coronavirus disease 2019, prevalence, death, prognosis

## Abstract

**Background:**

Arrhythmia is a very common complication of coronavirus disease 2019 (COVID-19); however, the prevalence of ventricular arrhythmia and associated outcomes are not well-explored. Here, we conducted a systematic review and meta-analysis to determine the prevalence and associated death of ventricular arrhythmia and sudden cardiac death (SCD) in patients with COVID-19.

**Methods:**

Databases of PubMed, Cochrane Library, Embase, and MdeRxiv were searched. Studies that could calculate the prevalence of ventricular arrhythmia/SCD during hospital admission or associated death in patients with COVID-19 were included. The study was registered with the PROSPERO (CRD42021271328).

**Results:**

A total of 21 studies with 13,790 patients were included. The pooled prevalence of ventricular arrhythmia was 5% (95% CI: 4–6%), with a relatively high-SCD prevalence (1.8% in hospitalized COVID-19 and 10% in deceased cases of COVID-19). Subgroup analysis showed that ventricular arrhythmia was more common in patients with elevated cardiac troponin T [ES (effect size): 10%, 95% CI: −0.2 to 22%] and in European (ES: 20%, 95% CI: 11–29%) populations. Besides, ventricular arrhythmia was independently associated with an increased risk of death in patients with COVID-19 [odds ratio (OR) = 2.83; 95% CI: 1.78–4.51].

**Conclusion:**

Ventricular arrhythmia and SCD resulted as a common occurrence with a high prevalence in patients with COVID-19 admitted to the hospital. Furthermore, ventricular arrhythmia significantly contributed to an increased risk of death in hospitalized patients with COVID-19. Clinicians might be vigilant of ventricular arrhythmias for patients with COVID-19, especially for severe cases.

**Systematic Review Registration:**

www.york.ac.uk/inst/crd, identifier: CRD42021271328.

## Introduction

Coronavirus disease 2019 (COVID-19) is a serious life-threatening disease caused by severe acute respiratory syndrome coronavirus 2 (SARS-CoV-2) infection, which first occurred in November 2019 (1), and then rapidly spread throughout the rest of the world. As of July 31, 2021, more than 198 million individuals were diagnosed with cases of COVID-19, exceeding 420 thousand deaths. Although COVID-19 is characterized by substantial respiratory pathology, several extrapulmonary manifestations, such as thrombotic complications, myocardial dysfunction, and arrhythmia, acute kidney injury are also commonly found in patients afflicted with the virus (2–4).

Cardiac arrhythmias, such as new-onset atrial fibrillation, heart block, and ventricular arrhythmias, are prevalent in the patients with COVID-19. An early report study on 138 patients from Wuhan, China, showed that 17% of the hospitalized patients suffered from total arrhythmia (5). Our recent meta-analysis also showed that the atrial fibrillation reached 10% and was associated with increased death in COVID-19 (6). Ventricular arrhythmia is still the major leading cause of death from the cardiovascular diseases (7). According to a multicenter cohort from the US, 6% of 4,250 patients with COVID-19 had prolonged QTc interval (corrected QT; >500 ms) at admission (8). This result suggested that COVID-19 might significantly contribute to an increased risk of ventricular tachycardia as QTc prolongation is believed to predispose to the ventricular arrhythmias associated with sudden death in certain cardiac diseases. Although several studies have reported the increased risk of ventricular tachycardia among patients with COVID-19, the exact prevalence of ventricular tachycardia in patients with COVID-19 remains unknown. Moreover, a case series also reported ventricular tachycardia and ventricular fibrillation as the primary cause of death in hospitalized patients with COVID-19 without a prior history of the structural heart disease (9). However, it remains unclear whether COVID-19 associated the ventricular arrhythmias are independently linked to increased death in the patients with COVID-19.

Furthermore, sudden cardiac death (SCD) is the most devastating manifestation of ventricular arrhythmias that has emerged as one of the disturbing concerns associated with the infection of COVID-19. Thus, we conducted a systematic review and meta-analysis to determine the prevalence and associated death of ventricular arrhythmia and SCD in patients with COVID-19.

## Methods

This study has been registered with PROSPERO (International prospective register of systematic reviews. www.york.ac.uk/inst/crd)-registration number-CRD42021271328. Furthermore, we conducted the meta-analysis according to the Preferred Reporting Items for Systematic Reviews and Meta-Analyses (PRISMA) Statement (2020) (Supplementary Table 1).

### Literature Search

The search was accomplished by two authors independently. PubMed, Embase, the Cochrane Library, and MedRxiv (https://www.medrxiv.org/) databases were searched mainly for the related studies up to July 21, 2021, without language restrictions. The search terms according to PICOS were as follows:

Population:

For COVID-19: “COVID-19” or “COVID-19 Virus Disease” or “COVID-19 Virus Infection” or “2019-nCoV Infection” or “Coronavirus Disease-19” or “2019 Novel Coronavirus Disease” or “2019 Novel Coronavirus Infection” or “2019-nCoV Disease” or “Coronavirus Disease 2019” or “SARS Coronavirus 2 Infection” or “SARS-CoV-2 Infection” or “COVID-19 Pandemic.”

Exposure:

For the ventricular arrhythmia: “ventricular arrhythmia” or “premature ventricular beats” or “ventricular ectopic beats” or “ventricular premature complex” or “premature ventricular contractions” or “ventricular tachycardia” or “ventricular tachyarrhythmia” or “ventricular flutter” or “ventricular fibrillation.”

For sudden cardiac death: “cardiac sudden death” or “sudden cardiac arrest” or “sudden cardiac death.”

Outcomes:

For death: “death” or “mortality.”

A detailed search strategy was described in Supplementary Table 2.

### Study Selection

All the results were organized by EndNote X9 software (Thomson Reuters, New York, NY, USA). After deleting the duplicate literature, the titles and abstracts were checked, and the relevant literature was preliminarily screened. Subsequently, full-texts of the relevant studies were searched to make sure they met the inclusion criteria. The inclusion criteria included the following: (1) studies that included adult patients diagnosed with COVID-19 based on the polymerase chain reaction tests; (2) studies that could calculate the prevalence of ventricular arrhythmia/SCD during hospital admission or reported the estimated effect between ventricular arrhythmia and death in patients with COVID-19.

If the same population was used in multiple studies, we selected the article with the most informative or the largest sample size. Studies that reported the effect of chloroquine/hydroxychloroquine and azithromycin in patients with COVID-19 were excluded because of the potential drug-induced ventricular arrhythmias risk. Certain publication types without sufficient data (reviews, meta-analysis, cases, editorials, and comments) were also excluded.

### Data Collection

The following information was independently abstracted by two researchers: the first author, publication year, country, time, study design, patient characteristics (sample size, age, and sex), number of ventricular arrhythmias, death, odds ratios (ORs), and the corresponding 95% CI and adjustments.

### Quality Assessment

For studies that reported the prevalence, the Joanna Briggs Institute (JBI) critical appraisal checklist was used to assess the study quality, where 0 score represented a failure to meet the requirements; 1 represented the lack of detailed description, 2 score represented detailed and comprehensive description. For studies that reported the association between ventricular arrhythmia and death in patients with COVID-19, the Newcastle-Ottawa Scale (NOS) was applied. Studies with scores of NOS ≥ 7 and JBI ≥ 14 were considered as high-quality researches (10).

### Statistical Analysis

RevMan software, version 5.3 (The Cochrane Collaboration 2014, Nordic Cochrane Center Copenhagen, Denmark) and Stata software (Version 14.0, Stata Corp LP, College Station, Texas, US) were both applied in our analysis. To explore the prevalence of ventricular arrhythmia and SCD in hospitalized patients with COVID-19, the exact binomial (Clopper–Pearson) method was used to calculate 95% CIs. Freeman–Tukey double arcsine transformation was used for standard estimates. To elucidate the outcome of ventricular arrhythmia and SCD in hospitalized patients with COVID-19, we pooled the ORs for each studies using the inverse variance method. We also estimated the adjusted ORs by calculating the natural logarithm of the OR (log [OR]) and its standard error (SElog [OR]), which is shown with 95% CIs. We evaluated the degree of heterogeneity using the *I*^2^ test (25, 50, and 75% represent low, moderate, and high heterogeneity). We used the random effect model in our study to improve the reliability.

Subgroup analyses were performed to study possible factors influencing our results, including ventricular arrhythmia, region, cardiac injury, and population. To ensure the reliability of study outcomes, we carried out sensitivity analyses by omitting each study in turn. *P* < 0.05 was considered statistically significant.

## Results

### Study Selection

**Figure 1:**
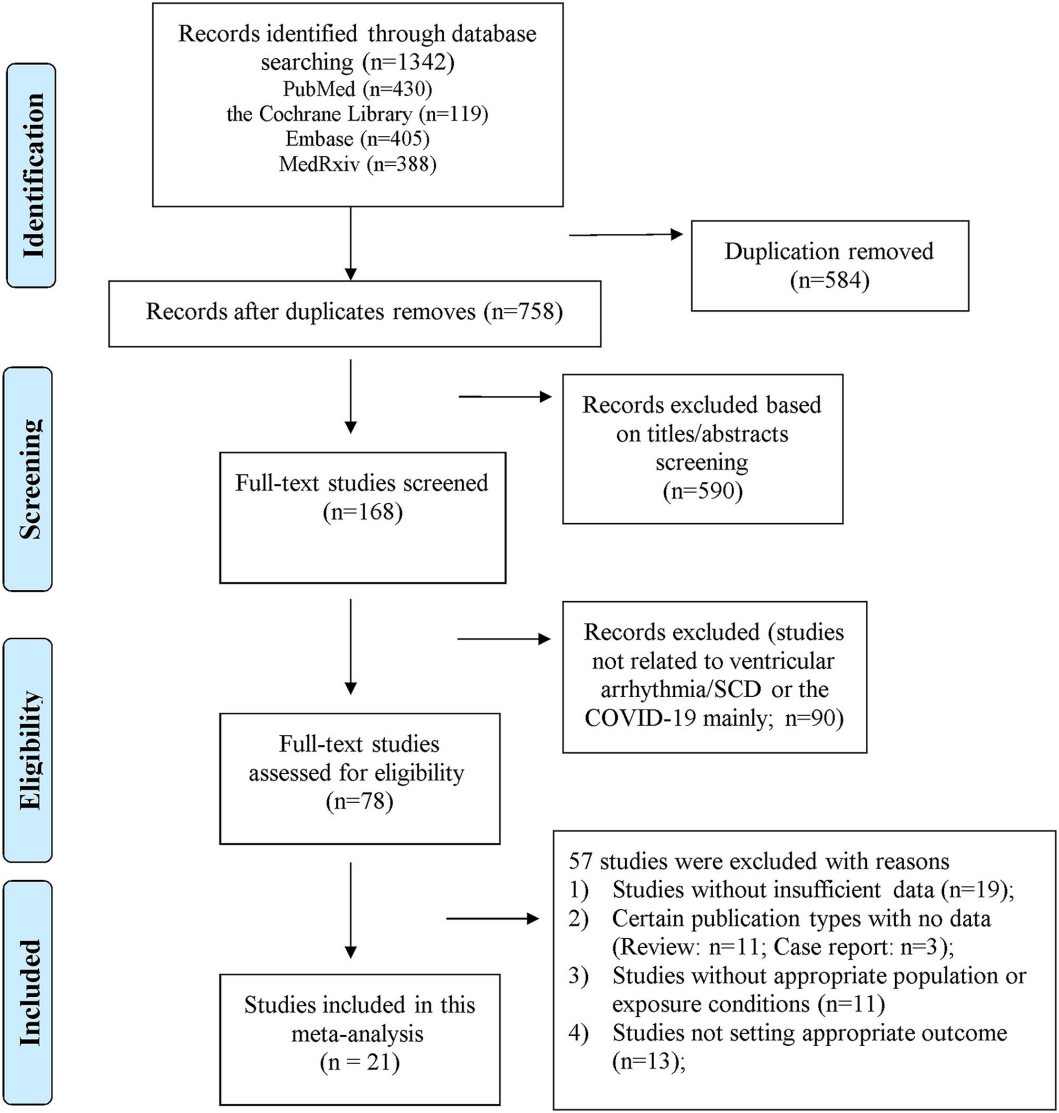
Flow chart of study selection.

The flow chart for the study selection process is shown in Figure 1. A total of 1,342 publications were identified following initial search (PubMed = 430; the Cochrane Library = 119; Embase = 405; MedRxiv = 388). After deleting 584 duplications and 680 irrelevant studies, the full-text assessment was performed on 78 studies. Subsequently, 57 articles were excluded due to the following reasons: (1) studies without insufficient data (*n* = 19); (2) certain publication types with no data (review = 11; case report = 3); (3) studies without appropriate population or exposure (*n* = 11); (4) studies that did not report target outcome (*n* = 13). Finally, 21 studies were included in the meta-analysis (11–31). All the excluded studies with reasons (*n* = 57) are shown in Supplementary Table 3.

### Study Characteristics and Quality

The basic characteristics of all the included articles are shown in Table 1. Twenty-one studies (11–31), which included 13,790 hospitalized patients with COVID-19 with a mean age ranging from 50 to 70.3 years, and with sample sizes ranging from 54 to 4,526, were published from 2020 to 2021 [eight of them published in 2021 (11, 15, 16, 19, 24–26, 31), others in 2020 (12–14, 17, 18, 20–23, 27–30)]. Nine reports were from Asia [eight from China (13, 16, 18, 21, 22, 28, 29, 31), one from Iran (19)], seven from USA (11, 12, 14, 15, 17, 25, 30), and five from Europe [three from Italy (20, 24, 27), one from Netherland (23), one from Germany (26)]. Besides, 15 of them were retrospective studies (11, 13, 15–19, 21–23, 27–31), 5 were prospective studies (12, 14, 20, 25, 26), and 1 was cross-section study (24).

**Table 1:** Basic characteristics of the articles included in the meta-analysis.

**Author, year, country**	**Study design**	**Diagnosis**	**Study populations**	**Total number**	**Male, age years**	**History of cardiovascular disease %**	**Medication %**	**Prevalence and endpoint reported**	**Cases/ Total**	**Adjusted effector (95% CI) and adjustments**
Antwi-Amoabeng et al. (11), USA	RC	ECG	Tertiary Care Hospital	186	53.2%, 60.0	CAD 3.2%; diabetes 37.1%; HF 9.7%; stroke 8.6%; hypertension 43.1%;	QT prolonging medications 57.5%	Premature ventricular complex	10/186	NA
Chen et al. (13), China	RC	ECG	NA	54	66.7%, 57.6	CAD 11.1%; diabetes 46.3%; hypertension 29.6%	NA	Ventricular tachycardia	3/54	NA
Cho et al. (14), USA	PC	Telemetry monitoring	Cedars-Sinai Medical Center	143	61.5%, 70.3	Diabetes 35.0%; CAD 18.9%; hypertension 55.2%; hyperlipidemia 41.3%; AF 12.6%	Azithromycin 59.4%; Tocilizumab 39.2%; Remdesivir 9.1%; HCQ 62.9%; Lopinavir/ritonavir 2.1%;	Premature ventricular complex Ventricular tachycardia Ventricular fibrillation SCD	143/41 24/143 1/143 1/143	NA
Coromilas et al. (15), USA	Case-control	ECG	Across the world for whom data was available	4,526	57.3%, 62.8	Diabetes 34.7%; CHF 16.9%; CAD 13.2%; AF/AFL 9.0%; VT 0.6%; stroke 6.1%; Vascular disease 3.7%; Hypertension 55%;	Azithromycin 49.8%; Antiviral 15.3%; IL-6 inhibitor 9.6%; Anticoagulation 29.4% HCQ 57.6%	Ventricular tachycardia Cardiac damage with ventricular arrhythmia	27/4,526 164/827	NA
Gao et al. (16), China	RC	ECG	Tongji Hospital	79	67.1%, 65.0	Diabetes 22.0%; CHF 3.0%; Cardiovascular disease 14.0%; hypertension 51.0%; stroke 13.0%	Glucocorticoid 77.0%; Antibiotics 67.0%; Anticoagulation 54.0%; Antiviral therapy 71.0%; Intravenous immunoglobulin 75.0%; Beta-blocker 16.0%; Tocilizumab 9.0%	Ventricular tachycardia Death	9/79	3.302 (1.524, 7.154) Clinical characteristics, comorbidities, laboratory indexes or therapies
Haji Aghajani et al. (19), Iran	RC	ECG	Imam-Hossein Hospital	893	55.3%, 61.8	NA	NA	Ventricular arrhythmia Death	28/893	1.854 (1.154, 2.979) Male sex, increase in age, sinus tachycardia, supraventricular arrhythmia, interventricular conduction delay, abnormal R wave progression, abnormal T wave
Lanza et al. (20), Italy	PC	ECG	Universita‘ Cattolica del Sacro Cuore Hospital	324	66.1%, 65.9	Known heart disease 20.7%; Hypertension 52.2%; diabetes 11.4%	NA	Premature ventricular complex	13/324	NA
Li et al. (21), China	Case-control	ECG	Renmin Hospital of Wuhan University	113	60.2%, 67.3	Hypertension 43.4%; Cardiovascular disease 20.4%; diabetes 18.6%;	Ribavirin 49.6%; Arborol 71.7%; Lopinavir/ritonavir 3.5%; HCQ 15.0%; Interferon α-2b injection 18.6%; Ganciclovir 17.7%; Oseltamivir 30.1%; Glucocorticoid 62.0%; Immunoglobulin 64.6%	Ventricular arrhythmia Premature ventricular complex Ventricular tachycardia Death	8/70 7/70 1/70	
										2.79 (1.11, 7.04) Age, initial neutrophil count, lactate dehydrogenase, C-reactive protein, immunoglobulin treatment, sinus tachycardia
Li et al. (22), China	Case-control	ICD	Wuhan Seventh People's Hospital	596	47.0%, 58.0	Diabetes 13.3%	Antivirus therapy 78.4%; Antibiotic therapy 74.8%; Glucocorticoid 29.5%; Immunoglobin 9.2%	Ventricular arrhythmia	12/596	NA
Linschoten et al. (23), Netherland	Case-control	ECG	CAPACITY-COVID (www.capacity-covid.eu)	3011	62.8%, 67.0	HF 5.3%; diabetes 23.1%; Hypertension 44.6%; Arrhythmia/conduction disorder 15.1%; CAD 5.3%; Valvular disease 4.3%	NA	Ventricular arrhythmia Cardia damage with ventricular arrhythmia	14/3011 14/349	NA
Malanchini et al. (24), Italy	Cross-sectional	Remote monitoring	Electrophysiology and Cardiac Pacing Unit at ASST Papa Giovanni XXIII Hospital	455	75.8%, 64.9	NA	Beta-blocker 85.9%; Amiodarone 34.2%; Mexiletine 3.5%	Ventricular arrhythmia Ventricular tachycardia Ventricular fibrillation	86/455 77/455 9/455	NA
Pareek et al. (25), USA	PC	ECG	Yale New Haven Hospital	586	47.4%, 67.0	Diabetes 38.5%; CAD 15.7%; Hypertension 58.1%; Cerebrovascular disease 9.3%; HF/cardiomyopathy 14.3%; AF/AFL10.1%; PAD 3.0%; Ventricular arrhythmia 1.4%	Beta blocker 27.4%; ACE inhibitor/ARB 31.9%; Aspirin 29.0%; Anticoagulant 11.1%; Antiarrhythmic 2.6%; CCB 24.0%;	Ventricular arrhythmia SCD Death	12/586 21/586	
										18.97 (3.68, 97.88) Age, sex, history of heart failure, history of ventricular arrhythmias, P2Y12 inhibitors, oxygen therapy at admission, and respiratory rates. CRP, albumin, and troponin T
Parwani et al. (26), Germany	PC	ECG	University Hospital Center at the Charité Berlin	113	73.5%, 64.1	CAD 18.6%; arrhythmias 15.9%; congestive HF 11.5%; hypertension 61.1%; AF/AFL 14.2%; ventricular tachycardia 0.9%	Beta blocker 27.4%; calcium antagonists 15.9%; ACEi/ARB/ARNI 34.5%; platelet inhibitor 23.9%;	Ventricular arrhythmia Premature ventricular complex Ventricular tachycardia Ventricular fibrillation	64/113 28/113 34/113 2/113	NA
Russo et al. (27), Italy	Case-control	ECG	Emergency Department of 10 Italian Hospitals	414	61.1%, 66.9	Diabetes 25.6%; AF 17.4%; HF 11.1%; stroke 8.4%; Hypertension 63.5%	ACEi/ARB 41.1%; beta-blocker 14.0%; Ca^2+^ antagonist 24.2%	Ventricular tachycardia Death	14/414	2.55 (1.5, 3.35) Male, age, hypertension, heart failure, chronic kidney disease, coronary artery disease
Shao et al. (28), China	Case-control	ECG	West Campus of Union Hospital in Wuhan	136	66.2%, 69.0	Hypertension 30.2%; diabetes 19.9%; Coronary heart disease 11.0%; cerebrovascular disease 3.7%	NA	Ventricular arrhythmia SCD	8/136 151/761	NA
Bhatla et al. (12), USA	PC	NA	Hospital of the University of Pennsylvania	700	45.0%; 50.0	Coronary heart disease 11.0%; hypertension 50.0%; HF 13.0%; diabetes 26.0% AF 6.0%	Hydroxychloroquine 25.0%; remdesivir 8.0%	Ventricular tachycardia SCD	10/700 9/700	NA
Gopinathannair et al. (17), USA	Case-control	NA	The Heart Rhythm Society (HRS) study	683	NA; NA	NA	Hydroxychloroquine/ chloroquine 33.5%; HCQ/chloroquine + azithromycin 31.0%	Ventricular tachycardia Premature ventricular complex Ventricular arrhythmia	93/683 60/683 33/683	NA
Guo et al. (18), China	Case-control	ECG	The Seventh Hospital of Wuhan City	187	48.7%; 58.5	Hypertension 32.6%; coronary heart disease 11.2%; cardiomyopathy 4.3%; diabetes 15.0%	Antivirus 88.8%; antibiotic 97.9%; glucocorticoid 56.7%; immune globulin 11.2%	Ventricular arrhythmia	11/187	NA
Si et al. (29), China	RC	ECG	Tongji Hospital in Wuhan	170	54.7%; 61.5	Hypertension 55.9%; diabetes 21.8%;stroke 3.5%	Antiviral 97.6%; antibiotic 95.9%; QT-prolonging medication 74.7%	Ventricular arrhythmia	1/170	NA
Turagam et al. (30), USA	RC	ECG/telemetry	Mount Sinai Hospital	140	72.9%; 61.0	Diabetes 39.0%; CAD 25.0%; Hypertension 61.0%; congestive HF 16.0%; Ventricular arrhythmias 1.0%; Atrial arrhythmia 14.0%;	Azithromycin 44.0%; remdesivir 1.0%; sorolumab 5.0%; tocilizumab 8.0%; glucocorticoid 5.0%; anticoagulation 18.0%; hydroxychloroquine 76.0%; antiarrhythmics 11.0%	Ventricular arrhythmia	7/140	NA
Yang et al. (31), China	RC	NA	Tongji Hospital	281	68.0%; 69.0	Hypertension 38.8%; diabetes 14.2%; CHD 11.4%	Antiviral 44.8%; antibiotic 96.8%; corticosteroid 89.7%; immune globulin 58.0%	SCD	28/281	NA

*ECG, electrocardiogram; CAD, coronary artery disease; HF, heart failure; AF, atrial fibrillation; AFL, atrial flutter; CHF, congestive heart failure; SCD, sudden cardiac death; VT, ventricular tachycardia; ACEi, angiotensin-converting enzyme inhibitor; ARB, angiotensin II receptor blocker; NA, not application; AHA COVID-19 CVD, the American Heart Association COVID-19 cardiovascular disease; CCB, calcium channel blocker; CRP, C reactive protein; RC, retrospective cohort; PC, prospective cohort; COVID-19, coronavirus disease 19*.

All studies (11–31) scored between 16 and 20 on the Joanna Briggs Institute Critical Appraisal Checklist, which meant these articles took rigorous methodology. In addition, five studies (16, 19, 21, 25, 27) involved the association between ventricular arrhythmia and death in patients with COVID-19, with NOS scores > 7, thus were regarded as moderate high-quality studies (Supplementary Table 4).

### The Prevalence of Ventricular Arrhythmia and SCD in Patients With COVID-19

**Figure 2:**
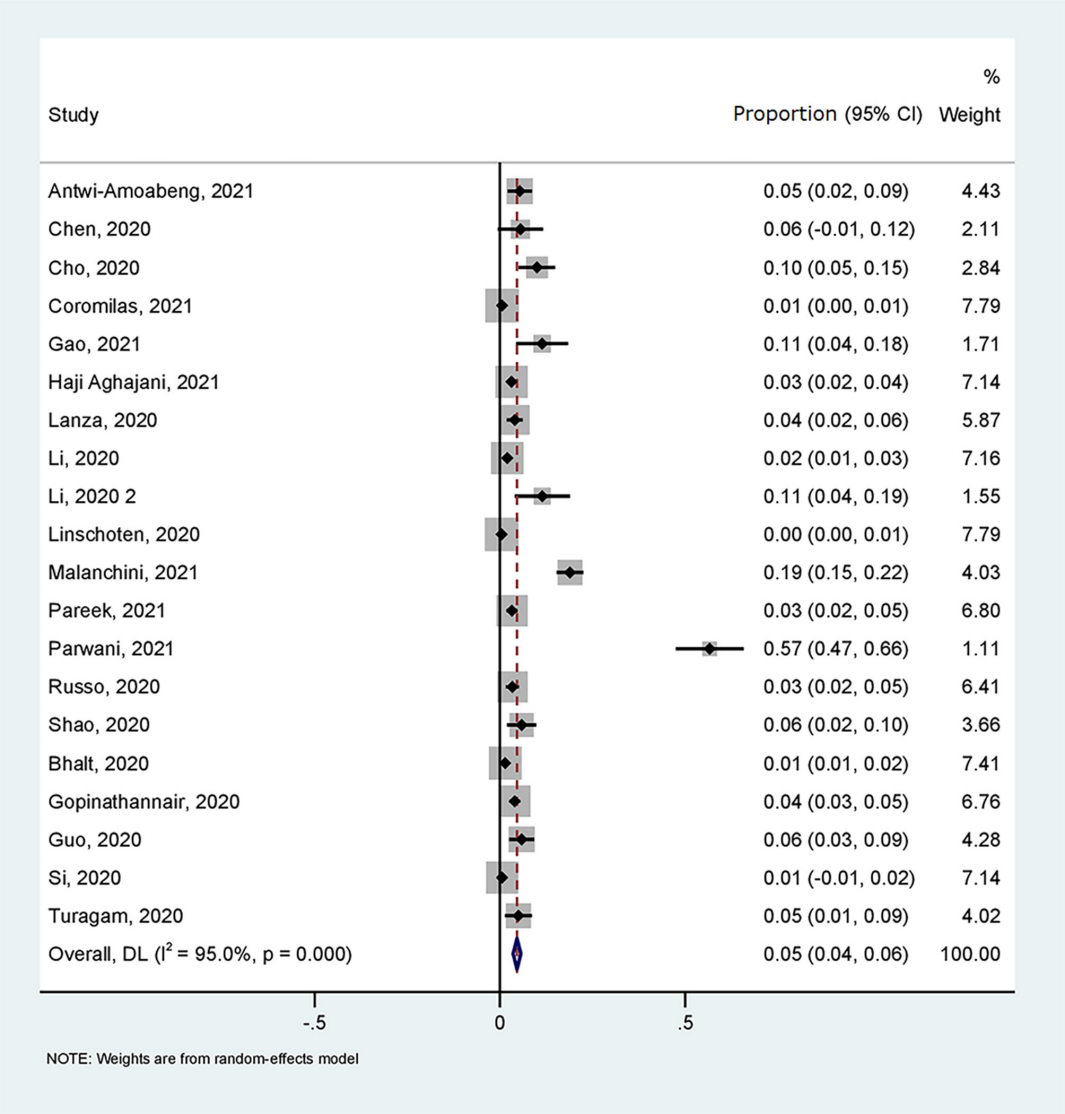
Forest plot for the prevalence of the ventricular arrhythmia in patients with COVID-19.

A total of 20 (11–30) studies with 13,509 patients reported the prevalence of ventricular arrhythmia in patients with COVID-19. As shown in Figure 2, the pooled prevalence of the ventricular arrhythmia was 5% (95% CI: 4–6%).

**Figure 3:**
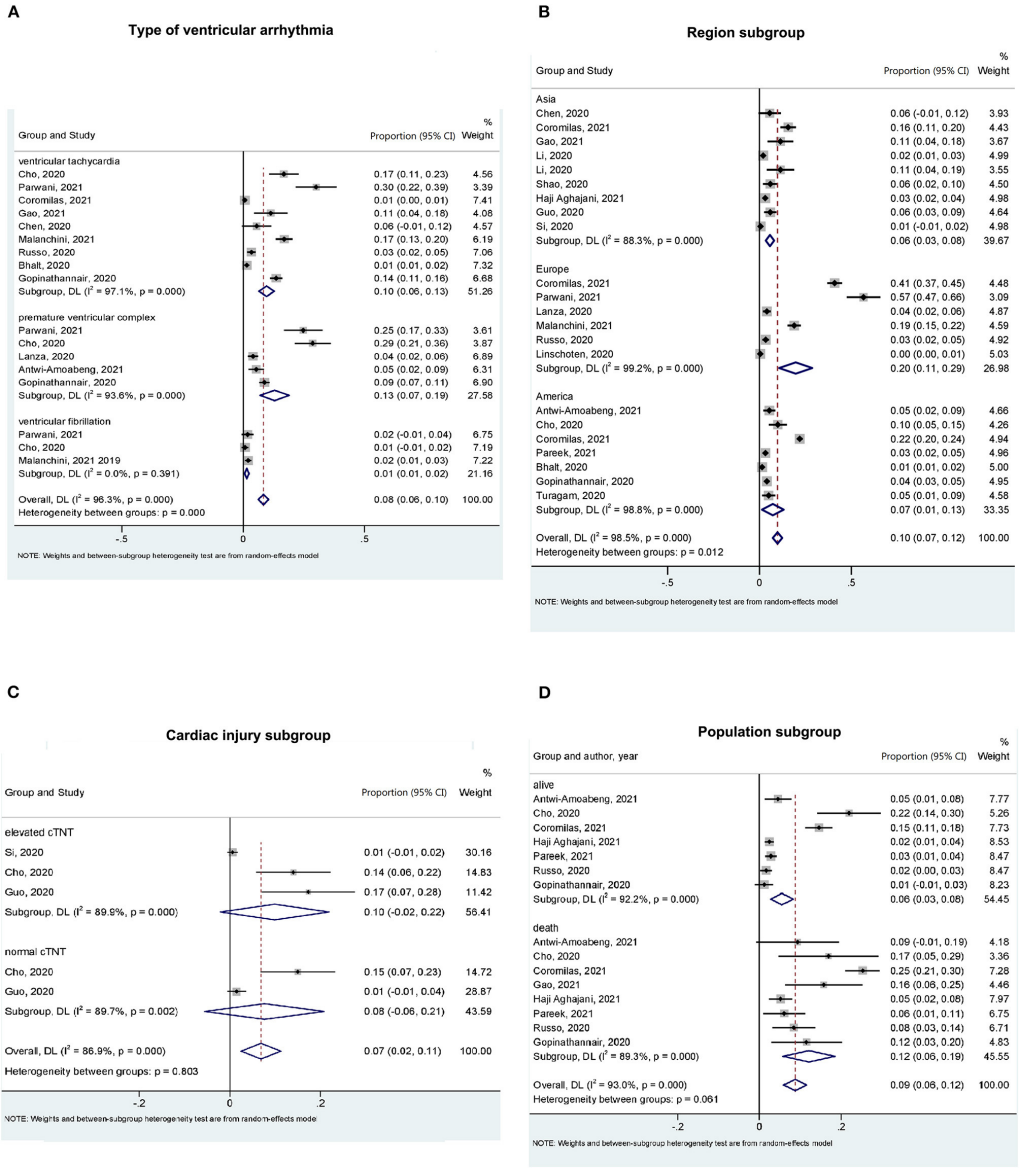
Subgroup analysis for the prevalence of ventricular arrhythmia in patients with COVID-19. **(A)** Ventricular arrhythmia type subgroup. According to ventricular arrhythmia type, ventricular tachycardia, premature ventricular complex, and ventricular fibrillation group were divided. **(B)** Region subgroup. According to region, Asia, Europe, and the United States group were divided. **(C)** Cardiac injury (cardiac troponin T level) subgroup. All the researches were divided into elevated cTNT and normal cTNT group according to cTNT level. **(D)** Population (alive and dead) subgroup. Alive and death population were divided.

Subgroup analysis showed that premature ventricular complex was the most common type in patients with COVID-19 (ES: 13%; 95% CI: 7–19%), followed by ventricular tachycardia (ES: 10%; 95% CI: 6–13%) and ventricular fibrillation (ES: 1%; 95% CI: 1–2%) (Figure 3A). According to area subgroup analysis, Europe had the highest prevalence (ES: 20%; 95% CI: 11–29%), followed by the United States (ES: 7%; 95% CI: 1–13%), while the lowest prevalence was found in Asia (ES: 6%; 95% CI: 3–8%) (Figure 3B). Furthermore, the prevalence of the ventricular arrhythmia in hospitalized patients with elevated cardiac troponin T was 1.25-fold higher than that without elevated cardiac troponin T (ES: 10 vs. 8%) (Figure 3C). The prevalence of ventricular arrhythmia in living and deceased hospitalized patients with COVID-19 was 6 and 12%, respectively (Figure 3D).

Two articles reported the prevalence of SCD in hospitalized patients with COVID-19 (28, 31). Article of Shao reported that the prevalence of SCD was 1.8% in the population with COVID-19 (28). Also, according to Yang et al. there was a higher SCD prevalence (10%) in the deceased population with COVID-19 (31).

### The Impact of Ventricular Arrhythmia on All-Cause Death With COVID-19

**Figure 4:**
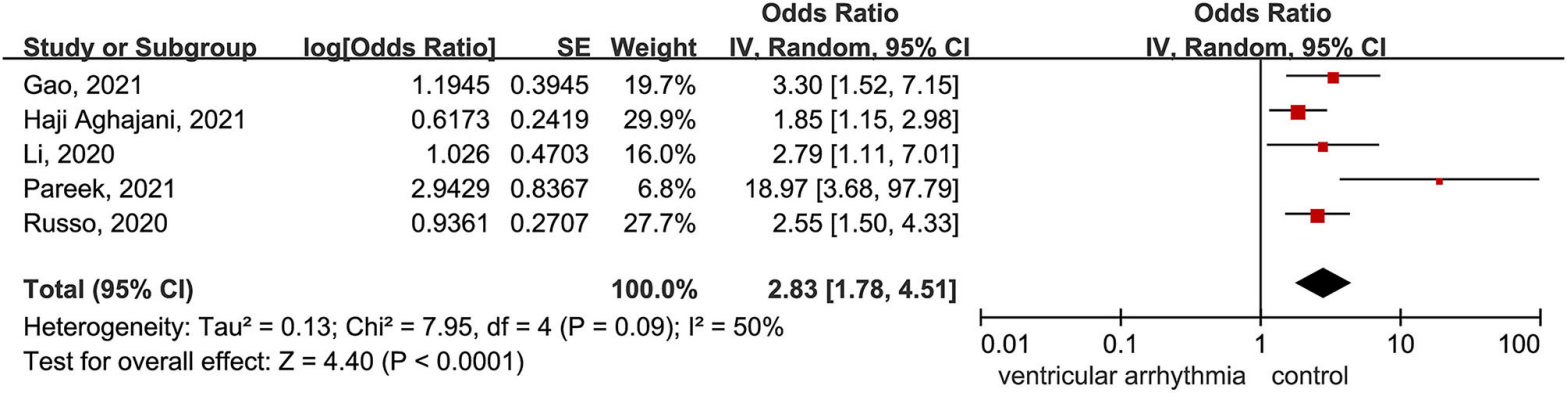
Forest plot for the association between the ventricular arrhythmia and death in patients with COVID-19.

Five multivariable-adjusted publications with 2,568 patients were included in the analysis (16, 19, 21, 25, 27). The results showed a positive association between ventricular arrhythmia and risk of death in hospitalized patients with COVID-19 (OR = 2.83; 95% CI: 1.78–4.51%; *I*^2^ = 50%) (Figure 4), revealing a moderate heterogeneity. These results were stable when excluding Pareek et al. (25) with no evidence of heterogeneity (OR = 2.35; 95% CI: 1.74–2.39%; *I*^2^ = 0%). After deleting each study in turn, sensitivity analyses indicated that our results were stable, with a range from 2.35 (95% CI: 1.74–3.19%) to 3.41 (95% CI: 1.94–6.00%) (Supplementary Figure 1).

### Publication Bias

The potential publication bias for death was not performed due to the limited number of studies (*N* < 10).

## Discussion

In this study, we pooled data from 21 studies with 13,790 hospitalized patients with COVID-19, finding that: (i) the prevalence of ventricular arrhythmia in hospitalized patients with COVID-19 was 5%. Specifically, the premature ventricular complex, ventricular tachycardia, and ventricular fibrillation occurred in 13, 10, 1% in the hospitalized patients, respectively. (ii) Ventricular arrhythmia was independently related to an increased risk of death in hospitalized patients with COVID-19. Overall, the ventricular arrhythmia and SCD were not uncommon and were associated with adverse outcomes in the hospitalized patients. To the best of our knowledge, this is the first meta-analysis that reported the prevalence of ventricular arrhythmia, SCD, and associated prognosis in the hospitalized patients with COVID-19.

Cardiac arrhythmia was identified as one of the major complications of SARS-COV during the outbreak in China in 2003 (32). Tachycardia was the most common type of arrhythmia among the patients infected with SARS-COV and was independent of fever (33, 34). Similarly, in hospitalized patients with COVID, arrhythmic events are not uncommon among the COVID-19 related cardiovascular complications. As we previously reported, atrial fibrillation is the most prevalent arrhythmia in hospitalized patients (6). Regarding ventricular arrhythmia, evidence from earliest cohorts from Wuhan, China, showed that ventricular arrhythmia occurred in 7% of patients (13/187) and that the rate of ventricular arrhythmia was almost doubled in patients with elevated troponin T levels on ICU admission (18), which is consistent with our results (Figure 3). However, the exact pathophysiology underlying ventricular arrhythmia in COVID-19 may be multifactorial and remains elusive.

First, as we previously described, cardiac injury or myocarditis commonly occurs due to the inhibited activity of angiotensin-converting enzyme 2 (ACE2) by SARS-COV2, which was found in 19% of hospitalized patients with COVID-19, and has been estimated to double among those with pre-existing cardiovascular and non-cardiovascular diseases (e.g., diabetes, hypertension, and cancers). These comorbidities might make their cardiomyocytes more vulnerable to be attacked by SARS-COV2 and thus causing a higher incidence of cardiac injury (35, 36). This cardiac injury might contribute to the abnormalities in cardiac electrophysiology, eventually inducing ventricular arrhythmia. This supposition is consistent with our subgroup analysis, which showed that the incident rate of ventricular arrhythmia increased with cardiac injury. Secondly, it is well-known that COVID-19 is characterized by the dysregulated immune response and cytokine release syndrome. Numerous studies have shown an elevation of serum inflammatory markers, such as C-reactive protein, ferritin, erythrocyte sedimentation rate (37). It has also been identified as a predictor of severity and death in patients with COVID-19. On the other hand, various pro-inflammatory factors, such as C-reactive protein and tumor necrosis factor α, have also been shown to promote ventricular arrhythmia significantly (38). Third, thrombotic complications are the main extrapulmonary manifestations of COVID-19 (37). For example, autopsies performed at a single academic medical center revealed deep venous thrombosis in 7 out of 12 patients (58%) who were not suspected of venous thromboembolism before death. Pulmonary embolism was the direct cause of death in four of these patients (39).

Limited studies reported the incidence of SCD in patients with COVID-19, our review showed it to be 1.8% in the all-hospitalized population with COVID-19 and 10% in the decreased patients. This incident rate is strikingly higher than that reported in the US in patients without COVID-19 (40). One study reported that the incidence of SCD was 14.9–110.8 per 100,000 in population with a non-COVID-19 in different regions (41). Nonetheless, the above results were consistent with several reports. For example, a cohort from Pennsylvania reported a 1.3% incidence of cardiac arrest amongst 700 urban patients admitted for COVID-19 (12). Furthermore, a multicenter cohort study in the US showed that 2.2% of non-ICU (intensive care unit) patients developed in-hospital sudden arrest (42). In the context of COVID-19, Acharya et al. showed the incidence of in-hospital sudden arrest for ICU patients was 15.4% in hospital patients (42). Yet, only ~7% of the patients with COVID-19 survived to discharge after experiencing in-hospital sudden arrest according to their report (42). Currently, the reason for this high SCD or cardiac arrest rate is not fully evident, and both the cardiac (e.g., undetected ventricular fibrillation) or non-cardiac (e.g., missed pulmonary embolisms) factors might be responsible for this condition.

Our results also showed that ventricular arrhythmia was more likely to occur in American and European patients compared to Asian patients. This should be interpreted considering the limited sample size and differences in baseline characteristics. Notably, the hospitalized American and European patients were mostly older compared with Chinese patients (Table 1). Additionally, the prevalence of common comorbidities, such as obesity and diabetes, was also higher in American and European populations than in Asia. All the aforementioned risk factors might be contributing to a higher incidence rate of cardiac injury or severity of COVID-19 cases in American and European populations. Therefore, the regional difference should be validated by further studies.

### Compassion With the Previous Study

Previous studies have proved that COVID-19 can significantly affect the cardiovascular system of the patient, leading to serious cardiovascular diseases (3, 4, 43). Also, the previous studies had revealed a positive relationship between arrhythmia and COVID-19 (4, 24, 43, 44). Two meta-analyses studied the relationship between COVID-19 and ventricular arrhythmia. However, one of them focused on the patients after chloroquine or hydroxychloroquine treatment (45), while another explored the effect of COVID-19 on QTd, Tp-e/QTc ratio, and Tp-e interval (46). In addition, two meta-analyses described the relationship between COVID-19 and all types of arrhythmias (47, 48). Nevertheless, most of the included studies reported atrial fibrillation. Our meta-analysis extended the previous study and quantified'prevalence of ventricular arrhythmias and is associated with the clinical outcomes in hospitalized patients with COVID-19.

### Clinical Implication

Considering the prevalence of the ventricular arrhythmias in patients with COVID-19, clinicians should be vigilant of ventricular arrhythmias in patients with COVID-19. Screening of high-risk groups for ventricular arrhythmias should be performed at admission. ECG monitoring at admission is suggested for those hospitalized patients who might be at higher risk for the cardiac arrhythmias, such as those with the cardiac injury, palpitations, dizziness, unexplained syncope, and prolonged QTc. In addition, although it is still being debated whether hydroxychloroquine and azithromycin are linked to increased risk of ventricular arrhythmia (49), hydroxychloroquine, and azithromycin can significantly prolong QT interval, which might lead to the ventricular arrhythmia (50, 51). The non-pharmacological treatment (e.g., nutrition support) might also benefit (51). Therefore, these aforementioned treatments might be more carefully evaluated before application or avoided for patients with COVID-19 who were susceptible to ventricular arrhythmias.

### Limitation

Our study has several limitations. First, a high degree of heterogeneity was observed in our results, which might be due to study design of the patients and baseline characteristics. For example, ventricular arrhythmia was monitored by a telemetry monitor in the study of Cho (14), while other studies used regular ECG or electrocardiography monitoring. Second, all the studies included hospitalized patients, which may overestimate the prevalence of the ventricular arrhythmia and its clinical impact on patients with COVID-19 compared with the community patients. Third, due to data restrictions, we could not explore the sex or age differences in the association between the ventricular arrhythmia and death. Third, as shown in Table 1, a number of patients receiving drugs tend to prolong the QT interval, which might overestimate the prevalence of the ventricular arrhythmia or SCD. Finally, considering the limited sample size, the incident rate related to the regional differences still needs to be validated by further studies.

## Conclusion

Ventricular arrhythmia and SCD resulted as a common occurrence with a high prevalence in the hospitalized patients with COVID-19. Furthermore, the ventricular arrhythmia significantly contributed to an increased risk of death in hospitalized patients with COVID-19. Clinicians might be vigilant of ventricular arrhythmias for patients with COVID-19, especially for the severe cases.

## Data Availability Statement

The original contributions presented in the study are included in the article/Supplementary Material, further inquiries can be directed to the corresponding authors.

## Author Contributions

XL and PY were responsible for the entire project and revised the draft. ZT, KM, and ML performed the data extraction, statistical analysis, drafted the first version of the manuscript, and interpreting the data. All authors participated in the interpretation of the results and prepared the final version of the manuscript.

## Funding

This work was supported in part by the National Natural Science Foundation of China (XL: 82100347, PY: 81760050 and 81760048), China Postdoctoral Science Foundation (XL: 2021M703724), and the Jiangxi Provincial Natural Science Foundation for Youth Scientific Research (PY: 20192ACBL21037).

## Conflict of Interest

The authors declare that the research was conducted in the absence of any commercial or financial relationships that could be construed as a potential conflict of interest.

## Publisher's Note

All claims expressed in this article are solely those of the authors and do not necessarily represent those of their affiliated organizations, or those of the publisher, the editors and the reviewers. Any product that may be evaluated in this article, or claim that may be made by its manufacturer, is not guaranteed or endorsed by the publisher.
