# Supplementary material for: The Prevalence and Associated Death of Ventricular Arrhythmia and Sudden Cardiac Death in Hospitalized Patients With COVID-19: A Systematic Review and Meta-Analysis
Source: Front Cardiovasc Med. 2022 Jan 21;8:795750. doi: 10.3389/fcvm.2021.795750 (PMC8814312; doi:10.3389/fcvm.2021.795750)

| **Section and Topic** | **Item #** | **Checklist item** | **Location where item is reported** |
| --- | --- | --- | --- |
| **TITLE** | | |  |
| Title | 1 | Identify the report as a systematic review. | 1 |
| **ABSTRACT** | | |  |
| Abstract | 2 | See the PRISMA 2020 for Abstracts checklist. | 2 |
| **INTRODUCTION** | | |  |
| Rationale | 3 | Describe the rationale for the review in the context of existing knowledge. | 3 |
| Objectives | 4 | Provide an explicit statement of the objective(s) or question(s) the review addresses. | 3-4 |
| **METHODS** | | |  |
| Eligibility criteria | 5 | Specify the inclusion and exclusion criteria for the review and how studies were grouped for the syntheses. | 5 |
| Information sources | 6 | Specify all databases, registers, websites, organisations, reference lists and other sources searched or consulted to identify studies. Specify the date when each source was last searched or consulted. | 5-6 |
| Search strategy | 7 | Present the full search strategies for all databases, registers and websites, including any filters and limits used. | 5-6 |
| Selection process | 8 | Specify the methods used to decide whether a study met the inclusion criteria of the review, including how many reviewers screened each record and each report retrieved, whether they worked independently, and if applicable, details of automation tools used in the process. | 6 |
| Data collection process | 9 | Specify the methods used to collect data from reports, including how many reviewers collected data from each report, whether they worked independently, any processes for obtaining or confirming data from study investigators, and if applicable, details of automation tools used in the process. | 6-7 |
| Data items | 10a | List and define all outcomes for which data were sought. Specify whether all results that were compatible with each outcome domain in each study were sought (e.g. for all measures, time points, analyses), and if not, the methods used to decide which results to collect. | 6-7 |
|  | 10b | List and define all other variables for which data were sought (e.g. participant and intervention characteristics, funding sources). Describe any assumptions made about any missing or unclear information. | 7 |
| Study risk of bias assessment | 11 | Specify the methods used to assess risk of bias in the included studies, including details of the tool(s) used, how many reviewers assessed each study and whether they worked independently, and if applicable, details of automation tools used in the process. | 7 |
| Effect measures | 12 | Specify for each outcome the effect measure(s) (e.g. risk ratio, mean difference) used in the synthesis or presentation of results. | 7 |
| Synthesis methods | 13a | Describe the processes used to decide which studies were eligible for each synthesis (e.g. tabulating the study intervention characteristics and comparing against the planned groups for each synthesis (item #5)). | 6-7 |
|  | 13b | Describe any methods required to prepare the data for presentation or synthesis, such as handling of missing summary statistics, or data conversions. | 6-7 |
|  | 13c | Describe any methods used to tabulate or visually display results of individual studies and syntheses. | 6-7 |
|  | 13d | Describe any methods used to synthesize results and provide a rationale for the choice(s). If meta-analysis was performed, describe the model(s), method(s) to identify the presence and extent of statistical heterogeneity, and software package(s) used. | 6-7 |
|  | 13e | Describe any methods used to explore possible causes of heterogeneity among study results (e.g. subgroup analysis, meta-regression). | 6-7 |
|  | 13f | Describe any sensitivity analyses conducted to assess robustness of the synthesized results. | 6-7 |
| Reporting bias assessment | 14 | Describe any methods used to assess risk of bias due to missing results in a synthesis (arising from reporting biases). | 7 |
| Certainty assessment | 15 | Describe any methods used to assess certainty (or confidence) in the body of evidence for an outcome. | 7 |
| **RESULTS** | | |  |
| Study selection | 16a | Describe the results of the search and selection process, from the number of records identified in the search to the number of studies included in the review, ideally using a flow diagram. | 7 |
|  | 16b | Cite studies that might appear to meet the inclusion criteria, but which were excluded, and explain why they were excluded. | 7 |
| Study characteristics | 17 | Cite each included study and present its characteristics. | 7 |
| Risk of bias in studies | 18 | Present assessments of risk of bias for each included study. | 7 |
| Results of individual studies | 19 | For all outcomes, present, for each study: (a) summary statistics for each group (where appropriate) and (b) an effect estimate and its precision (e.g. confidence/credible interval), ideally using structured tables or plots. | 7-9 |
| Results of syntheses | 20a | For each synthesis, briefly summarise the characteristics and risk of bias among contributing studies. | 7-9 |
|  | 20b | Present results of all statistical syntheses conducted. If meta-analysis was done, present for each the summary estimate and its precision (e.g. confidence/credible interval) and measures of statistical heterogeneity. If comparing groups, describe the direction of the effect. | 7-9 |
|  | 20c | Present results of all investigations of possible causes of heterogeneity among study results. | 7-9 |
|  | 20d | Present results of all sensitivity analyses conducted to assess the robustness of the synthesized results. | 7-9 |
| Reporting biases | 21 | Present assessments of risk of bias due to missing results (arising from reporting biases) for each synthesis assessed. | 7-9 |
| Certainty of evidence | 22 | Present assessments of certainty (or confidence) in the body of evidence for each outcome assessed. | 7-9 |
| **DISCUSSION** | | |  |
| Discussion | 23a | Provide a general interpretation of the results in the context of other evidence. | 9-13 |
|  | 23b | Discuss any limitations of the evidence included in the review. | 9-13 |
|  | 23c | Discuss any limitations of the review processes used. | 9-13 |
|  | 23d | Discuss implications of the results for practice, policy, and future research. | 9-13 |
| **OTHER INFORMATION** | | |  |
| Registration and protocol | 24a | Provide registration information for the review, including register name and registration number, or state that the review was not registered. | 13-14 |
|  | 24b | Indicate where the review protocol can be accessed, or state that a protocol was not prepared. | 13-14 |
|  | 24c | Describe and explain any amendments to information provided at registration or in the protocol. | 13-14 |
| Support | 25 | Describe sources of financial or non-financial support for the review, and the role of the funders or sponsors in the review. | 13-14 |
| Competing interests | 26 | Declare any competing interests of review authors. | 13-14 |
| Availability of data, code and other materials | 27 | Report which of the following are publicly available and where they can be found: template data collection forms; data extracted from included studies; data used for all analyses; analytic code; any other materials used in the review. | 13-14 |

*From:*  Page MJ, McKenzie JE, Bossuyt PM, Boutron I, Hoffmann TC, Mulrow CD, et al. The PRISMA 2020 statement: an updated guideline for reporting systematic reviews. BMJ 2021;372:n71. doi: 10.1136/bmj.n71

For more information, visit: <http://www.prisma-statement.org/>

**Table S2:** Detailed description of the search strategy

| **For ventricular arrhythmia** | |
| --- | --- |
| **PubMed** | |
| #1 | ventricular arrhythima [MeSH Terms] |
| #2 | ‘ventricular arrhythmia’ OR ‘premature ventricular beats’ OR ‘ventricular ectopic beats’ OR ‘ventricular premature complex’ OR ‘premature ventricular contractions’ OR ‘ventricular tachycardia’ OR ‘ventricular tachyarrhythmia’ OR ‘ventricular flutter’ OR ‘ventricular fibrillation’ |
| #3 | COVID-19 [MeSH Terms] |
| #4 | ‘COVID-19’ OR ‘COVID-19 Virus Disease’ OR ‘COVID-19 Virus Infection’ OR ‘2019-nCoV Infection’ OR ‘Coronavirus Disease-19’ OR ‘2019 Novel Coronavirus Disease’ OR ‘2019 Novel Coronavirus Infection’ OR ‘2019-nCoV Disease’ OR ‘Coronavirus Disease 2019’ OR ‘SARS Coronavirus 2 Infection’ OR ‘SARS-CoV-2 Infection’ OR ‘COVID-19 Pandemic’ |
| #5 | #1 OR #2 |
| #6 | #3 OR #4 |
| #7 | #5 AND #6 |
| **Embase** | |
| #1 | ‘ventricular arrhythmia’:ab,ti |
| #2 | ‘ventricular arrhythmia’ OR ‘premature ventricular beats’ OR ‘ventricular ectopic beats’ OR ‘ventricular premature complex’ OR ‘premature ventricular contractions’ OR ‘ventricular tachycardia’ OR ‘ventricular tachyarrhythmia’ OR ‘ventricular flutter’ OR ‘ventricular fibrillation’ |
| #3 | ‘COVID-19’:ab.ti |
| #4 | ‘COVID-19’ OR ‘COVID-19 Virus Disease’ OR ‘COVID-19 Virus Infection’ OR ‘2019-nCoV Infection’ OR ‘Coronavirus Disease-19’ OR ‘2019 Novel Coronavirus Disease’ OR ‘2019 Novel Coronavirus Infection’ OR ‘2019-nCoV Disease’ OR ‘Coronavirus Disease 2019’ OR ‘SARS Coronavirus 2 Infection’ OR ‘SARS-CoV-2 Infection’ OR ‘COVID-19 Pandemic’ |
| #5 | #1 OR #2 |
| #6 | #3 OR #4 |
| #7 | #5 AND #6 |
| **Cochrane** | |
| #1 | ‘ventricular arrhythmia’:ti, ab, kw |
| #2 | ‘ventricular arrhythmia’ OR ‘premature ventricular beats’ OR ‘ventricular ectopic beats’ OR ‘ventricular premature complex’ OR ‘premature ventricular contractions’ OR ‘ventricular tachycardia’ OR ‘ventricular tachyarrhythmia’ OR ‘ventricular flutter’ OR ‘ventricular fibrillation’ |
| #3 | ‘COVID-19’:ti, ab, kw |
| #4 | ‘COVID-19’ OR ‘COVID-19 Virus Disease’ OR ‘COVID-19 Virus Infection’ OR ‘2019 nCoV Infection’ OR ‘Coronavirus Disease-19’ OR ‘2019 Novel Coronavirus Disease’ OR ‘2019 Novel Coronavirus Infection’ OR ‘2019 nCoV Disease’ OR ‘Coronavirus Disease 2019’ OR ‘SARS Coronavirus 2 Infection’ OR ‘SARS-CoV-2 Infection’ OR ‘COVID-19 Pandemic’ |
| #5 | #1 OR #2 |
| #6 | #3 OR #4 |
| #7 | #5 AND #6 |
| **For SCD** | |
| **PubMed** | |
| #1 | SCD [MeSH Terms] |
| #2 | ‘cardiac sudden death’ OR ‘sudden cardiac arrest’ OR ‘sudden cardiac death’ |
| #3 | COVID-19 [MeSH Terms] |
| #4 | ‘COVID-19’ OR ‘COVID-19 Virus Disease’ OR ‘COVID-19 Virus Infection’ OR ‘2019-nCoV Infection’ OR ‘Coronavirus Disease-19’ OR ‘2019 Novel Coronavirus Disease’ OR ‘2019 Novel Coronavirus Infection’ OR ‘2019-nCoV Disease’ OR ‘Coronavirus Disease 2019’ OR ‘SARS Coronavirus 2 Infection’ OR ‘SARS-CoV-2 Infection’ OR ‘COVID-19 Pandemic’ |
| #5 | #1 OR #2 |
| #6 | #3 OR #4 |
| #7 | #5 AND #6 |
| **Embase** | |
| #1 | ‘SCD’:ab,ti |
| #2 | ‘cardiac sudden death’ OR ‘sudden cardiac arrest’ OR ‘sudden cardiac death’ |
| #3 | ‘COVID-19’:ab.ti |
| #4 | ‘COVID-19’ OR ‘COVID-19 Virus Disease’ OR ‘COVID-19 Virus Infection’ OR ‘2019-nCoV Infection’ OR ‘Coronavirus Disease-19’ OR ‘2019 Novel Coronavirus Disease’ OR ‘2019 Novel Coronavirus Infection’ OR ‘2019-nCoV Disease’ OR ‘Coronavirus Disease 2019’ OR ‘SARS Coronavirus 2 Infection’ OR ‘SARS-CoV-2 Infection’ OR ‘COVID-19 Pandemic’ |
| #5 | #1 OR #2 |
| #6 | #3 OR #4 |
| #7 | #5 AND #6 |
| **Cochrane** | |
| #1 | ‘SCD’:ti, ab, kw |
| #2 | ‘cardiac sudden death’ OR ‘sudden cardiac arrest’ OR ‘sudden cardiac death’ |
| #3 | ‘COVID-19’:ti, ab, kw |
| #4 | ‘COVID-19’ OR ‘COVID-19 Virus Disease’ OR ‘COVID-19 Virus Infection’ OR ‘2019 nCoV Infection’ OR ‘Coronavirus Disease-19’ OR ‘2019 Novel Coronavirus Disease’ OR ‘2019 Novel Coronavirus Infection’ OR ‘2019 nCoV Disease’ OR ‘Coronavirus Disease 2019’ OR ‘SARS Coronavirus 2 Infection’ OR ‘SARS-CoV-2 Infection’ OR ‘COVID-19 Pandemic’ |
| #5 | #1 OR #2 |
| #6 | #3 OR #4 |
| #7 | #5 AND #6 |

**Table S3:** Studies excluded (n=57) with reasons

| Studies excluded | Reasons |
| --- | --- |
| Abrams, 2020^1^ | Not target outcome: arrhythmia |
| Andrade, 2020^2^ | Not interest data |
| Acharya, 2021 ^3^ | Not target outcome: cardiac arrest |
| Awwab, 2021^4^ | Not target outcome: bradyarrhythmia |
| Berman, 2020^5^ | Not target exposure: after treatment |
| Bhatla, 2020^6^ | Not target outcome: ICU |
| Carretta, 2021^7^ | Review |
| Chinitz, 2020^8^ | Case report |
| Cipriani, 2020^9^ | Not interest data |
| Clark, 2020^10^ | Not target outcome: other major events |
| De Vita, 2020^11^ | Not interest data |
| Desai, 2021^12^ | Review |
| Doyen, 2021^13^ | Not target population: patients with cardiac injury |
| Dutta, 2021^14^ | Not target relationship: drug and arrhythmia |
| Ece, 2021^15^ | Not interest data |
| Elsaid, 2020^16^ | Case report |
| Gasperetti, 2020^17^ | Not target exposure: after treatment |
| Goldman, 2021^18^ | Not interest data |
| Gopinathannair, 2020^19^ | Not interest data |
| Guzik, 2020^20^ | Review |
| Hayek, 2020^21^ | Not target population: cardiopulmonary resuscitation |
| Hoang, 2021^22^ | Not target outcome: bradyarrhythmia |
| Hu, 2020^23^ | Review |
| Jain, 2020^24^ | Not interest data |
| Jankowska, 2020^25^ | Review |
| Koc, 2020^26^ | Not interest data |
| Long, 2021^27^ | Review |
| Maneikis, 2021^28^ | Not interest data |
| Manolis, 2020^29^ | Review |
| Matteucci, 2021^30^ | Not target relationship: home management and arrhythmia |
| Mercedes, 2021^31^ | Not target population: pregnant |
| Mohammad, 2020^32^ | Review |
| Moschini, 2021^33^ | Not target exposure: drug using |
| Nemer, 2021^34^ | Not interest data |
| Niazi, 2021^35^ | Review |
| Özdemir, 2021^36^ | Not target exposure: after treatment |
| Öztürk, 2021^37^ | Not interest data |
| Rav-Acha, 2021^38^ | Not target outcome: atrial fibrillation |
| Raza, 2021^39^ | Review |
| Rivinius, 2020^40^ | Not target population: after cardiac transplant |
| Sala, 2020^41^ | Not target outcome |
| Samuel, 2020^42^ | Not interest data |
| Santoro, 2020^43^ | Not interest data |
| Sarayani, 2021^44^ | Not target exposure: after treatment |
| Sassone, 2021^45^ | Not target population: patients with cardioverter defibrillator |
| Si, 2020^46^ | Not target relationship: ventricular arrhythmias and biomarker level |
| Song, 2020^47^ | Not interest data |
| Sperotto, 2021^48^ | Case report |
| Tondas, 2021^49^ | Not target outcome: atrioventricular block |
| Turagam, 2020^50^ | Not target outcome: arrhythmias |
| Valverde, 2021^51^ | Not target population: children |
| Wang, 2020^52^ | Not target relationship: arrhythmia and biomarkers |
| Wong, 2021^53^ | Not interest data |
| Yamin, 2020^54^ | Review |
| Yenerçağ, 2020^55^ | Not interest data |
| Zeng, 2020^56^ | Not target outcome: arrhythmia |
| Zhou, 2021^57^ | Not target outcome: sinus bradycardia |

1. Abrams MP, Wan EY, Waase MP, et al. Clinical and cardiac characteristics of COVID-19 mortalities in a diverse New York City Cohort. *J Cardiovasc Electrophysiol* 2020; 31: 3086-3096. 2020/10/07. DOI: 10.1111/jce.14772.

2. Andrade JA, Muzykovsky K and Truong J. Risk factors for mortality in COVID-19 patients in a community Teaching Hospital. *Open Forum Infectious Diseases* 2020; 7: S268. Conference Abstract. DOI: 10.1093/ofid/ofaa417.594.

3. Acharya P, Ranka S, Sethi P, et al. Incidence, Predictors, and Outcomes of In-Hospital Cardiac Arrest in COVID-19 Patients Admitted to Intensive and Non-Intensive Care Units: Insights From the AHA COVID-19 CVD Registry. *J Am Heart Assoc* 2021; 10: e021204. 2021/08/12. DOI: 10.1161/jaha.120.021204.

4. Awwab H, Solorzano J, Jaisingh K, et al. Cardiac pauses in critically ill Coronavirus Disease-2019 patients. *Heart and Mind* 2021; 5: 4-8. Original Article. DOI: 10.4103/hm.hm_35_20.

5. Berman JP, Abrams MP, Kushnir A, et al. Cardiac electrophysiology consultative experience at the epicenter of the COVID-19 pandemic in the United States. *Indian Pacing and Electrophysiology Journal* 2020; 20: 250-256. Article. DOI: 10.1016/j.ipej.2020.08.006.

6. Bhatla A, Mayer MM, Adusumalli S, et al. COVID-19 and cardiac arrhythmias. *Heart Rhythm* 2020; 17: 1439-1444. 2020/06/26. DOI: 10.1016/j.hrthm.2020.06.016.

7. Carretta DM, Silva AM, D'Agostino D, et al. Cardiac involvement in COVID-19 patients: A contemporary review. *Infectious Disease Reports* 2021; 13: 494-517. Review. DOI: 10.3390/idr13020048.

8. Chinitz JS, Goyal R, Harding M, et al. Bradyarrhythmias in patients with COVID-19: Marker of poor prognosis? *Pacing Clin Electrophysiol* 2020; 43: 1199-1204. 2020/08/22. DOI: 10.1111/pace.14042.

9. Cipriani A, Zorzi A, Ceccato D, et al. Arrhythmic profile and 24-hour QT interval variability in COVID-19 patients treated with hydroxychloroquine and azithromycin. *International Journal of Cardiology* 2020; 316: 280-284. Article. DOI: 10.1016/j.ijcard.2020.05.036.

10. Clark BC, Sanchez-De-toledo J, Bautista-Rodriguez C, et al. Cardiac abnormalities seen in pediatric patients during the SARS-COV2 pandemic: An international experience. *Journal of the American Heart Association* 2020; 9. Article. DOI: 10.1161/JAHA.120.018007.

11. De Vita A, Ravenna SE, Covino M, et al. Electrocardiographic Findings and Clinical Outcome in Patients with COVID-19 or Other Acute Infectious Respiratory Diseases. *J Clin Med* 2020; 9 2020/11/18. DOI: 10.3390/jcm9113647.

12. Desai AD, Boursiquot BC, Melki L, et al. Management of Arrhythmias Associated with COVID-19. *Current Cardiology Reports* 2021; 23. Review. DOI: 10.1007/s11886-020-01434-7.

13. Doyen D, Dupland P, Morand L, et al. Characteristics of Cardiac Injury in Critically Ill Patients With Coronavirus Disease 2019. *Chest* 2021; 159: 1974-1985. 2020/11/02. DOI: 10.1016/j.chest.2020.10.056.

14. Dutta S, Kaur R, Bhardwaj P, et al. Hydroxychloroquine as therapeutic option in covid-19: Analysis of suspected cardiovascular adverse drug events reported in the vigibase. *Bangladesh Journal of Medical Science* 2021; 20: 897-910. Article. DOI: 10.3329/bjms.v20i4.54150.

15. Ece İ, Koçoğlu M, Kavurt AV, et al. Assessment of Cardiac Arrhythmic Risk in Children With Covid-19 Infection. *Pediatr Cardiol* 2021; 42: 264-268. 2020/10/03. DOI: 10.1007/s00246-020-02474-0.

16. Elsaid O, McCullough PA, Tecson KM, et al. Ventricular Fibrillation Storm in Coronavirus 2019. *American Journal of Cardiology* 2020; 135: 177-180. Article. DOI: 10.1016/j.amjcard.2020.08.033.

17. Gasperetti A, Biffi M, Duru F, et al. Arrhythmic safety of hydroxychloroquine in COVID-19 patients from different clinical settings. *Europace* 2020; 22: 1855-1863. Article. DOI: 10.1093/europace/euaa216.

18. Goldman A, Bomze D, Dankner R, et al. Cardiovascular adverse events associated with hydroxychloroquine and chloroquine: A comprehensive pharmacovigilance analysis of pre-COVID-19 reports. *Br J Clin Pharmacol* 2021; 87: 1432-1442. 2020/09/24. DOI: 10.1111/bcp.14546.

19. Gopinathannair R, Merchant FM, Lakkireddy DR, et al. COVID-19 and cardiac arrhythmias: a global perspective on arrhythmia characteristics and management strategies. *Journal of Interventional Cardiac Electrophysiology* 2020; 59: 329-336. Article. DOI: 10.1007/s10840-020-00789-9.

20. Guzik TJ, Mohiddin SA, Dimarco A, et al. COVID-19 and the cardiovascular system: Implications for risk assessment, diagnosis, and treatment options. *Cardiovascular Research* 2020; 116: 1666-1687. Review. DOI: 10.1093/cvr/cvaa106.

21. Hayek SS, Brenner SK, Azam TU, et al. In-hospital cardiac arrest in critically ill patients with covid-19: multicenter cohort study. *Bmj* 2020; 371: m3513. 2020/10/02. DOI: 10.1136/bmj.m3513.

22. Hoang L, Wang L, Sidhu M, et al. BRADYARRHYTHMIAS AND MORTALITY IN COVID-19 PATIENTS. *Journal of the American College of Cardiology* 2021; 77: 3080-3080. DOI: doi:10.1016/S0735-1097(21)04435-1.

23. Hu TY, Lee JZ and Asirvatham SJ. Cardiovascular considerations in coronavirus disease 2019 with a special focus on arrhythmia. *Journal of Innovations in Cardiac Rhythm Management* 2020; 11. Review. DOI: 10.19102/icrm.2020.110804.

24. Jain S, Workman V, Ganeshan R, et al. Enhanced electrocardiographic monitoring of patients with Coronavirus Disease 2019. *Heart Rhythm* 2020; 17: 1417-1422. Article. DOI: 10.1016/j.hrthm.2020.04.047.

25. Jankowska EA, Sierpiński R, Tkaczyszyn M, et al. Chloroquine and hydroxychloroquine for the prevention and therapy of coronavirus disease 2019: new hopes and old cardiovascular concerns. *Kardiologia Polska* 2020; 78: 811-817. Review. DOI: 10.33963/KP.15511.

26. Koc M, Sumbul HE, Gulumsek E, et al. Disease Severity Affects Ventricular Repolarization Parameters in Patients With COVID-19. *Arq Bras Cardiol* 2020; 115: 907-913. 2020/12/10. DOI: 10.36660/abc.20200482.

27. Long B, Brady WJ, Bridwell RE, et al. Electrocardiographic manifestations of COVID-19. *American Journal of Emergency Medicine* 2021; 41: 96-103. Review. DOI: 10.1016/j.ajem.2020.12.060.

28. Maneikis K, Ringeleviciute U, Bacevicius J, et al. Mitigating arrhythmia risk in Hydroxychloroquine and Azithromycin treated COVID-19 patients using arrhythmia risk management plan. *IJC Heart and Vasculature* 2021; 32. Article. DOI: 10.1016/j.ijcha.2020.100685.

29. Manolis AS, Manolis AA, Manolis TA, et al. COVID-19 infection and cardiac arrhythmias. *Trends in Cardiovascular Medicine* 2020; 30: 451-460. Review. DOI: 10.1016/j.tcm.2020.08.002.

30. Matteucci A, Bonanni M, Centioni M, et al. Home management of heart failure and arrhythmias in patients with cardiac devices during pandemic. *Journal of Clinical Medicine* 2021; 10. Article. DOI: 10.3390/jcm10081618.

31. Mercedes BR, Serwat A, Naffaa L, et al. New-onset myocardial injury in pregnant patients with coronavirus disease 2019: a case series of 15 patients. *Am J Obstet Gynecol* 2021; 224: 387.e381-387.e389. 2020/10/26. DOI: 10.1016/j.ajog.2020.10.031.

32. Mohammad FS, Karmakar V and Irfan Z. Hydroxychloroquine and azithromycin combination could be lethal to COVID-19 patients. *Farmacia* 2020; 68: 384-389. Review. DOI: 10.31925/farmacia.2020.3.2.

33. Moschini L, Loffi M, Regazzoni V, et al. Effects on QT interval of hydroxychloroquine associated with ritonavir/darunavir or azithromycin in patients with SARS-CoV-2 infection. *Heart and Vessels* 2021; 36: 115-120. Article. DOI: 10.1007/s00380-020-01671-4.

34. Nemer DM, Wilner BR, Burkle A, et al. Clinical Characteristics and Outcomes of Non-ICU Hospitalization for COVID-19 in a Nonepicenter, Centrally Monitored Healthcare System. *Journal of hospital medicine* 2021; 16: 7-14. Article. DOI: 10.12788/jhm.3510.

35. Niazi I and Khan M. Arrhythmias in the age of coronavirus disease 2019. *Journal of Innovations in Cardiac Rhythm Management* 2021; 12: 4345-4348. Note. DOI: 10.19102/ICRM.2021.120107.

36. Özdemir İH, Özlek B, Özen MB, et al. Hydroxychloroquine/azithromycin treatment, QT interval and ventricular arrhythmias in hospitalised patients with COVID-19. *International Journal of Clinical Practice* 2021; 75. Article. DOI: 10.1111/ijcp.13896.

37. Öztürk F, Karaduman M, Çoldur R, et al. Interpretation of arrhythmogenic effects of COVID-19 disease through ECG. *Aging Male* 2021; 23: 1362-1365. Article. DOI: 10.1080/13685538.2020.1769058.

38. Rav-Acha M, Orlev A, Itzhaki I, et al. Cardiac arrhythmias amongst hospitalised Coronavirus 2019 (COVID-19) patients: Prevalence, characterisation, and clinical algorithm to classify arrhythmic risk. *International Journal of Clinical Practice* 2021; 75. Article. DOI: 10.1111/ijcp.13788.

39. Raza HA, Tariq J, Agarwal V, et al. COVID-19, hydroxychloroquine and sudden cardiac death: implications for clinical practice in patients with rheumatic diseases. *Rheumatology International* 2021; 41: 257-273. Review. DOI: 10.1007/s00296-020-04759-2.

40. Rivinius R, Kaya Z, Schramm R, et al. COVID-19 among heart transplant recipients in Germany: a multicenter survey. *Clin Res Cardiol* 2020; 109: 1531-1539. 2020/08/13. DOI: 10.1007/s00392-020-01722-w.

41. Sala S, Peretto G, De Luca G, et al. Low prevalence of arrhythmias in clinically stable COVID-19 patients. *Pacing Clin Electrophysiol* 2020; 43: 891-893. 2020/06/17. DOI: 10.1111/pace.13987.

42. Samuel S, Friedman RA, Sharma C, et al. Incidence of arrhythmias and electrocardiographic abnormalities in symptomatic pediatric patients with PCR-positive SARS-CoV-2 infection, including drug-induced changes in the corrected QT interval. *Heart Rhythm* 2020; 17: 1960-1966. Article. DOI: 10.1016/j.hrthm.2020.06.033.

43. Santoro F, Monitillo F, Raimondo P, et al. QTc interval prolongation and life-threatening arrhythmias during hospitalization in patients with COVID-19. Results from a multi-center prospective registry. *Clinical infectious diseases : an official publication of the Infectious Diseases Society of America* 2020. Article in Press. DOI: 10.1093/cid/ciaa1578.

44. Sarayani A, Cicali B, Henriksen CH, et al. Safety signals for QT prolongation or Torsades de Pointes associated with azithromycin with or without chloroquine or hydroxychloroquine. *Res Social Adm Pharm* 2021; 17: 483-486. 2020/04/25. DOI: 10.1016/j.sapharm.2020.04.016.

45. Sassone B, Virzì S, Bertini M, et al. Impact of the COVID-19 lockdown on the arrhythmic burden of patients with implantable cardioverter-defibrillators. *Pacing Clin Electrophysiol* 2021; 44: 1033-1038. 2021/05/23. DOI: 10.1111/pace.14280.

46. Si D, Du B, Ni L, et al. Death, discharge and arrhythmias among patients with COVID-19 and cardiac injury. *CMAJ* 2020; 192: E791-E798. Article. DOI: 10.1503/cmaj.200879.

47. Song L, Zhao S, Wang L, et al. Cardiovascular Changes in Patients With COVID-19 From Wuhan, China. *Front Cardiovasc Med* 2020; 7: 150. 2020/10/27. DOI: 10.3389/fcvm.2020.00150.

48. Sperotto F, Friedman KG, Son MBF, et al. Cardiac manifestations in SARS-CoV-2-associated multisystem inflammatory syndrome in children: a comprehensive review and proposed clinical approach. *Eur J Pediatr* 2021; 180: 307-322. 2020/08/18. DOI: 10.1007/s00431-020-03766-6.

49. Tondas AE, Mulawarman R, Trifitriana M, et al. Arrhythmia Risk Profile and Ventricular Repolarization Indices in COVID-19 Patients: A Systematic Review and Meta-Analysis. *J Infect Dev Ctries* 2021; 15: 224-229. 2021/03/11. DOI: 10.3855/jidc.13922.

50. Turagam MK, Musikantow D, Goldman ME, et al. Malignant Arrhythmias in Patients With COVID-19: Incidence, Mechanisms, and Outcomes. *Circulation Arrhythmia and electrophysiology* 2020; 13: e008920. Article. DOI: 10.1161/CIRCEP.120.008920.

51. Valverde I, Singh Y, Sanchez-de-Toledo J, et al. Acute Cardiovascular Manifestations in 286 Children With Multisystem Inflammatory Syndrome Associated With COVID-19 Infection in Europe. *Circulation* 2021; 143: 21-32. 2020/11/10. DOI: 10.1161/circulationaha.120.050065.

52. Wang Y, Wang Z, Tse G, et al. Cardiac arrhythmias in patients with COVID-19. *J Arrhythm* 2020; 36: 827-836. 2020/10/08. DOI: 10.1002/joa3.12405.

53. Wong AO, Gurung B, Wong WS, et al. Adverse effects of hydroxychloroquine and azithromycin on contractility and arrhythmogenicity revealed by human engineered cardiac tissues. *J Mol Cell Cardiol* 2021; 153: 106-110. 2020/12/30. DOI: 10.1016/j.yjmcc.2020.12.014.

54. Yamin M and Demili AU. Prevention of Ventricular Arrhythmia and Sudden Cardiac Death in COVID-19 Patients. *Acta medica Indonesiana* 2020; 52: 290-296. Review.

55. Yenerçağ M, Arslan U, Doğduş M, et al. Evaluation of electrocardiographic ventricular repolarization variables in patients with newly diagnosed COVID-19. *Journal of Electrocardiology* 2020; 62: 5-9. Article. DOI: 10.1016/j.jelectrocard.2020.07.005.

56. Zeng JH, Wu WB, Qu JX, et al. Cardiac manifestations of COVID-19 in Shenzhen, China. *Infection* 2020. Article in Press. DOI: 10.1007/s15010-020-01473-w.

57. Zhou M, Wong CK, Un KC, et al. Cardiovascular sequalae in uncomplicated COVID-19 survivors. *PLoS One* 2021; 16: e0246732. 2021/02/12. DOI: 10.1371/journal.pone.0246732.

**Table S4**. Quality assessment of included studies

| Author  (Publication Year) | JBI Critical Appraisal Checklist for Studies Reporting Prevalence Data | | | | | | | | | | | |
| --- | --- | --- | --- | --- | --- | --- | --- | --- | --- | --- | --- | --- |
|  | a | b | c | d | e | f | g | h | i | j | Total |  |
| Antwi-Amoabeng, 2021 | 2 | 2 | 0 | 2 | 2 | 2 | 2 | 2 | 1 | 2 | 17 |  |
| Chen, 2020 | 2 | 2 | 0 | 1 | 2 | 2 | 2 | 2 | 1 | 2 | 16 |  |
| Cho, 2020 | 2 | 2 | 0 | 2 | 2 | 2 | 2 | 2 | 2 | 2 | 18 |  |
| Coromilas, 2021 | 2 | 2 | 2 | 2 | 2 | 2 | 2 | 2 | 2 | 2 | 20 |  |
| Gao, 2021 | 2 | 2 | 0 | 2 | 2 | 2 | 2 | 2 | 2 | 2 | 18 |  |
| Haji Aghajani. 2021 | 2 | 2 | 2 | 2 | 2 | 2 | 2 | 2 | 2 | 2 | 20 |  |
| Lanza, 2020 | 2 | 2 | 2 | 2 | 2 | 2 | 2 | 2 | 1 | 2 | 19 |  |
| Li, 2020 | 2 | 2 | 2 | 2 | 2 | 2 | 2 | 2 | 2 | 2 | 20 |  |
| Li, 2020 | 2 | 2 | 0 | 2 | 2 | 2 | 2 | 2 | 1 | 2 | 17 |  |
| Linschoten, 2020 | 2 | 2 | 2 | 2 | 2 | 2 | 2 | 2 | 2 | 2 | 20 |  |
| Malanchini, 2021 | 2 | 2 | 2 | 2 | 2 | 2 | 2 | 2 | 2 | 2 | 20 |  |
| Pareek, 2021 | 2 | 2 | 2 | 2 | 2 | 2 | 2 | 2 | 2 | 2 | 20 |  |
| Parwani, 2021 | 2 | 2 | 0 | 2 | 2 | 2 | 2 | 2 | 2 | 2 | 18 |  |
| Russo, 2020 | 2 | 2 | 2 | 2 | 2 | 2 | 2 | 2 | 2 | 2 | 20 |  |
| Shao, 2020 | 2 | 2 | 0 | 2 | 2 | 2 | 2 | 2 | 1 | 2 | 17 |  |
| Bhalt, 2020 | 2 | 2 | 2 | 2 | 2 | 2 | 2 | 2 | 2 | 2 | 20 |  |
| Gopinathannair, 2020 | 2 | 2 | 2 | 1 | 2 | 2 | 2 | 2 | 2 | 2 | 19 |  |
| Guo, 2020 | 2 | 2 | 0 | 2 | 2 | 2 | 2 | 2 | 2 | 2 | 18 |  |
| Si, 2020 | 2 | 2 | 0 | 2 | 2 | 2 | 2 | 2 | 2 | 2 | 18 |  |
| Turagam, 2020 | 2 | 2 | 0 | 2 | 2 | 2 | 2 | 2 | 2 | 2 | 18 |  |
| Yang, 2021 | 2 | 2 | 0 | 2 | 2 | 2 | 2 | 2 | 2 | 2 | 18 |  |

a. Was the sample representative of target population?

b. Were study participants recruited in an appropriate way?

c. Was the sample size adequate?

d. Were the study subjects and the setting described in detail?

e. Was the data analysis conducted with sufficient coverage of the identified sample?

f. Were objective, standard criteria used for the measurement of the condition?

g. Was the condition measured reliably?

h. Was their appropriate statistical analysis?

i. Are all important confounding factors and subgroups differences identified and accounted for?

j. Were subpopulations identified using objective criteria?

| Author  (Publication Year) | Newcastle-Ottawa Scale | | | | | | | | | |
| --- | --- | --- | --- | --- | --- | --- | --- | --- | --- | --- |
|  | Selection | | | Comparability | | | Outcome | | | Total |
|  | a | b | c | d | e | f | g | h | i |  |
| Gao, 2021 | 1 | 1 | 1 | 1 | 1 | 1 | 1 | 1 | 0 | 8 |
| Haji Aghajani, 2021 | 1 | 1 | 1 | 1 | 1 | 1 | 1 | 1 | 0 | 8 |
| Li, 2020 | 1 | 1 | 1 | 1 | 1 | 1 | 1 | 1 | 0 | 8 |
| Pareek, 2021 | 1 | 1 | 1 | 1 | 1 | 1 | 1 | 1 | 0 | 8 |
| Russo, 2020 | 1 | 1 | 1 | 1 | 1 | 1 | 1 | 1 | 0 | 8 |

1. Representativeness of the exposed cohort.
2. Selection of the non-exposed cohort.
3. Ascertainment of exposure.
4. Demonstration that outcome of interest was not present at start of study.
5. Comparability of cohorts on the basis of the design or analysis (adjusted for age).
6. Comparability of cohorts on the basis of the design or analysis (adjusted for any other factor).
7. Assessment of outcome.
8. Was follow-up long enough for outcomes to occur.
9. Adequacy of follow-up of cohorts.

**Supplemental Figure 1**. Sensitivity analyses of association between ventricular arrhythmia and COVID-19-related death by omitting one study at once


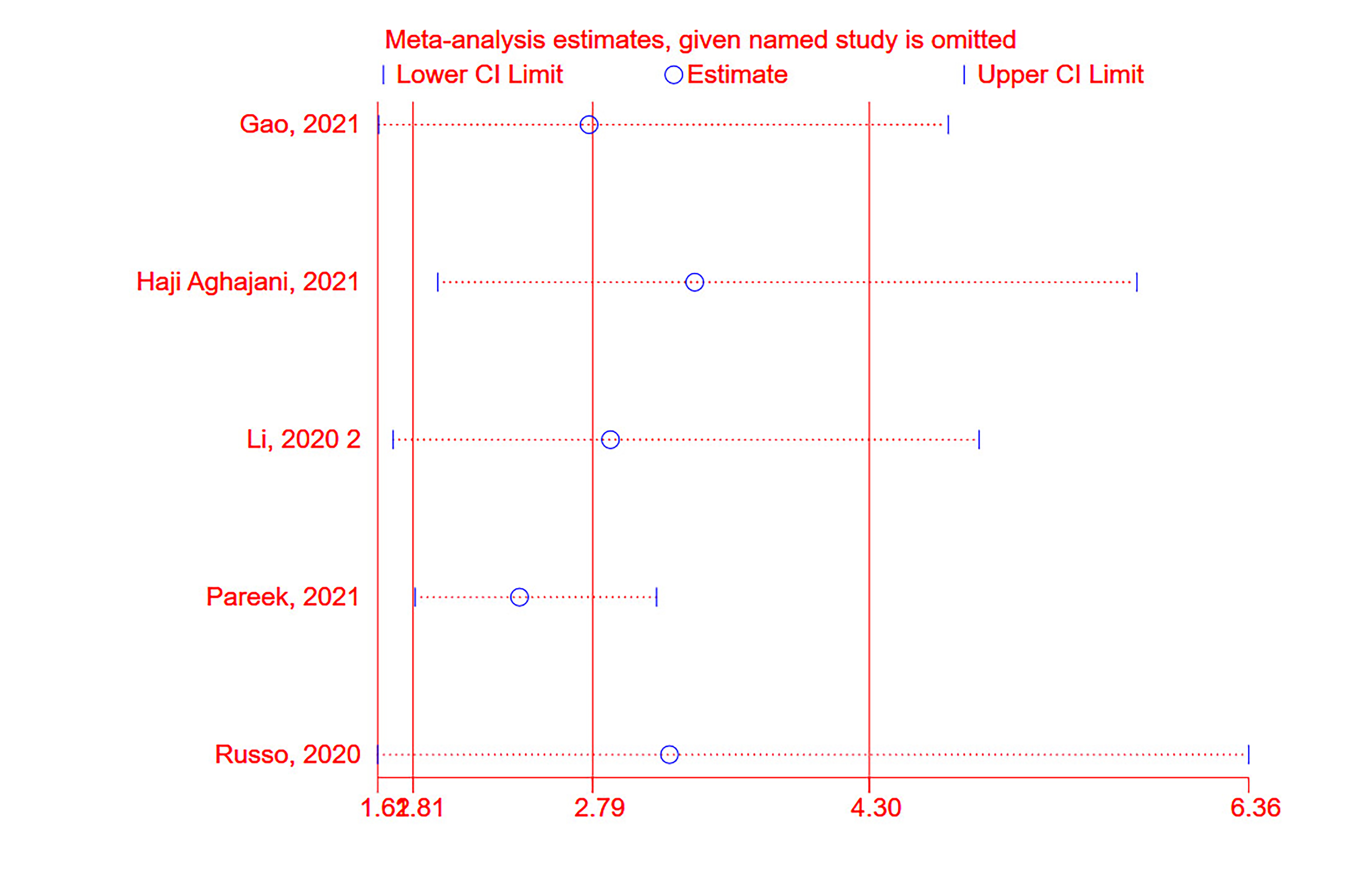

Supplement: Supplementary file 1 [file Data_Sheet_1.DOC]
